# FLLL31 Induces Apoptosis via the FOXO4/BCL6 Axis to Inhibit Bladder Cancer Progression

**DOI:** 10.1155/humu/5947612

**Published:** 2025-12-22

**Authors:** Yu Han, Jingxuan Peng, Wenjie Yan, Ziqiang Wu, Zhengyan Tang

**Affiliations:** ^1^ Department of Urology, Xiangya Hospital, Central South University, Changsha, China, csu.edu.cn; ^2^ Provincial Laboratory for Diagnosis and Treatment of Genitourinary System Disease, Changsha, China

**Keywords:** apoptosis, BCL6, bladder cancer, FLLL31, FOXO4

## Abstract

Bladder cancer represents one of the most common malignancies globally, posing a severe threat to human health. Through compound library screening, we identified tetramethylcurcumin (FLLL31), a diketone analog of curcumin, as exhibiting significant inhibitory effects on the malignant biological behaviors of bladder cancer cells. Although possessing diverse biological activities, the application of FLLL31 in bladder cancer has not been reported previously. To investigate the function and mechanism of FLLL31, we assessed its impact on the proliferation, migration, and invasion of T24 and 5637 cells using CCK‐8, EdU, colony formation, and Transwell. The in vivo efficacy of FLLL31 was evaluated by intraperitoneal injection in BALB/c‐nu mice bearing subcutaneous xenografts. Utilizing RNA‐seq, qRT‐PCR, Western blotting, electron microscopy, flow cytometry, and JC‐1 staining, we further explored the mechanism underlying FLLL31′s inhibition of malignant behaviors in bladder cancer cells. The results demonstrate that FLLL31 inhibits malignant bladder cancer behaviors by inducing apoptosis via the FOXO4/BCL6 axis. This pathway was further confirmed by the observation that lentiviral knockdown of either FOXO4 or BCL6 attenuated FLLL31‐induced apoptosis. Mechanistically, FLLL31 upregulates FOXO4, leading to increased BCL6 expression. This subsequently suppresses the antiapoptotic protein Bcl‐xL, thereby triggering apoptosis. These findings highlight the therapeutic potential of FLLL31 for bladder cancer and identify the FOXO4/BCL6 pathway as a promising novel target.

**Summary**

• In this study, we found that FLLL31 activates the mitochondria‐dependent apoptosis pathway by targeting the FOXO4/BCL6 axis to inhibit the malignant phenotype of bladder cancer cells, which provides a new drug candidate and target for the treatment of bladder cancer.

## 1. Introduction

Bladder cancer (BC) is one of the most common malignant tumors of the urinary tract [1]. Worldwide, the incidence rate of BC ranks ninth among malignant tumors, and the mortality rate ranks 13th [2, 3]. Although some progress has been made in the treatment of BC in recent years, the clinical efficacy is still not satisfactory [4]. Therefore, the development of novel therapeutic agents with both efficient antitumor activity and low toxicity is of great clinical significance. Tetramethylcurcumin (FLLL31) is a diketone analog of curcumin, in which the phenolic hydroxyl group has been replaced by a methyl group in the molecular structure [5]. Compared with standard curcumin, FLLL31 has been structurally modified to have a lower starting concentration, a higher stability, and a better performance under the same conditions than standard curcumin. FLLL31 is structurally modified to have a lower onset concentration, higher stability, and higher plasma concentration than standard curcumin under the same conditions [6, 7]. Currently, there are few studies on FLLL31, and its mechanism of action in BC has not been reported in the literature. Studies in tumors have mainly focused on their targeted inhibitory effect on the STAT3 signaling pathway, inhibiting STAT3 phosphorylation, DNA‐binding activity, and transcriptional activation to induce apoptosis in pancreatic cancer and breast cancer cells [5, 8]. Therefore, the present study was designed to investigate the potential biological function of FLLL31 in BC.

Forkhead Box O4 (FOXO4) belongs to the FOXO family of transcription factors (TFs) [9, 10], which plays an important role in the regulation of oncological diseases. It has been demonstrated that the mRNA level and protein expression of the FOXO4 gene in BC tissues are significantly lower than those in normal bladder tissues adjacent to cancer. Further analysis suggests that the expression level of FOXO4 is significantly correlated with the clinicopathological features and prognosis of BC, and its low expression may constitute an independent risk factor for poor prognosis of BC [11]. In addition to its direct effects, FOXO4 can influence tumorigenesis and therapy by indirectly affecting a wide range of cellular physiological activities [12].

B‐Cell Lymphoma 6 (BCL6) is a nuclear transcriptional repressor that can regulate tumor cell proliferation and apoptosis through gene translocation and deregulated expression [13]. Notably, FOXO4 can regulate BCL6 expression through multiple mechanisms, such as transcriptional repression mediated by direct binding to specific sequences in the promoter region of the BCL6 gene [14] or dephosphorylation and translocation to the nucleus, which induces apoptosis by inhibiting BCL6 expression [15].

Mitochondria‐dependent apoptosis is one of the major pathways of apoptosis [16], which is centrally characterized by the BCL2 family of proteins that regulate altered mitochondrial membrane permeability (MMP) and proapoptotic factor release through dynamic interactions [17, 18]. Bcl‐xL is one of the most important antiapoptotic members of the BCL2 family of proteins [19, 20], which are mainly localized in the mitochondrial outer membrane [21]. It prevents the activation of downstream effector proteins BAX and BAK by binding to and neutralizing the BH3 domain of proapoptotic proteins. BAX/BAK oligomerization is a key step in the formation of the mitochondrial membrane pore [22]; the release of proapoptotic factors, such as Cytochrome C; the triggering of caspase cascade reaction; and the execution of cell apoptosis [23]. Therefore, Bcl‐xL is the central gateway to prevent mitochondrial outer membrane permeabilization (MOMP) and apoptosis from occurring. This pathway is also central in tumorigenesis, and an imbalance in its regulatory network can cause tumor cells to evade apoptosis [24, 25]. In addition, Bcl‐xL‐expressing tumor cells are significantly less sensitive to mitochondria‐dependent apoptosis induced by chemotherapeutic agents such as paclitaxel [26] and cisplatin, which is an important mechanism for clinical failure.

## 2. Materials and Methods

### 2.1. Drugs and Pharmaceutical Preparations

FLLL31 (Selleck, China) was dissolved in DMSO (Aladdin, China) to 1 mM prior to use according to instructions. Subsequently, the stock solution was diluted to 100 *μ*M as the mother liquor using the complete culture solution, dispensed, and stored in a −20°C refrigerator for spare. For experimental use, 100 *μ*M of the mother liquor was taken and diluted to the corresponding concentration with the complete culture solution according to the experimental requirements.

### 2.2. Cell Line, Culture Condition, and Virus Transfection

Human bladder urothelial carcinoma (BLCA) cell lines T24 (RRID: CVCL_0554) and 5637 (RRID: CVCL_0126) were obtained from the Shanghai Cell Bank of the Chinese Academy of Sciences (Chinese Academy of Sciences, China) on May 20, 2022. The cell lines T24 and 5637 were authenticated using short tandem repeat (STR) profiling (Pricella Biotechnology, China), and their genetic profiles matched reference databases with an exact match value (EV) of 1.0 (T24) and 0.89 (5637), confirming their identity and absence of cross‐contamination. The date of the test was July 23, 2022. Mycoplasma testing was performed regularly, and all cultures were confirmed negative prior to experimental use. Cells were cultured in RPMI 1640 medium (Shanghai Yuanpei Bio‐technology Co., Shanghai, China) containing 10% fetal bovine serum (BI, The State of Israel) in a humidified environment at 37°C with 5% CO_2_. The same conditions were used for T24 shRNA knockdown cell lines. Lentiviral vectors were obtained from General Biol (Anhui, China). T24 cells were cultured in six‐well plates for 24 h and then transfected with FOXO4 knockdown lentiviral vector or BCL6 knockdown lentiviral vector. The transfection system was supplemented with 5 *μ*g/mL polybrene (Solarbio, China), and the medium was replaced with fresh medium after 24 h of culture. Infected cells were selected with puromycin (Beyotime, China). Human BLCA cell lines T24 and 5637 were free of mycoplasma contamination for the described experiments.

### 2.3. Cell Viability, Proliferation, Migration, and Invasion Function Assays

For cell viability, 5000 cells per well were inoculated in 96‐well plates. FLLL31 was added to the corresponding wells in a concentration gradient. The treatment time was 24 h. Then, CCK‐8 reagent (MCE, Shanghai, China) was added according to the instructions under the condition of light avoidance, and the OD value was detected at 450 nm to obtain the IC_50_. For the EdU cell proliferation assay, 5000 cells per well were inoculated in 96‐well plates, and 2.5/5 *μ*M FLLL31 was added, respectively (drug treatment time was 24 h). The IC_50_ was obtained by using BeyoClick EdU Cell Proliferation Kit with AF401 (MCE, Shanghai, China). In 96‐well plates, 2.5/5 *μ*M FLLL31 was added, respectively (drug treatment time 24 h), and BeyoClick EdU Cell Proliferation Kit with AF488 (Beyotime, China) was used to complete the experiments according to the instructions. Clone formation experiments were inoculated in 12‐well plates at a density of 500 cells per well, and 2.5/5 *μ*M FLLL31 was added, respectively (drug treatment time 24 h). Clones were cultured for 14 days and then stained using crystal violet. For Transwell migration experiments, Transwell chambers were used (Corning, United States), and the upper chamber was filled with cell suspensions diluted in serum‐free medium (1 × 10^6^/well). Medium containing 10% serum was added to the lower chamber, and FLLL31 at a final concentration of 2.5/5 *μ*M was added to the upper chamber, respectively (treatment time 24 h), and stained with crystal violet. For the Transwell invasion assay, the bottom membrane of the Transwell chambers was coated with a 1:8 dilution of 50 mg/L Matrigel (Corning, United States). The upper chamber surface of the membrane at the bottom of the Transwell was dried at 4°C for 2 h. Cell suspension diluted in serum‐free medium was added to the upper chamber (1 × 10^6^/well), and the lower chamber was filled with 10% serum‐containing medium. The final concentration of FLLL31 was added to the upper chamber with a final concentration of 2.5/5 *μ*M (treatment time: 24 h), and the cells were stained with crystal violet staining, respectively.

### 2.4. Animal Experimentation

Five‐week‐old *BALB/c-nu* mice were provided by Jiangsu Collective Pharmachem Biotechnology Co. (Nanjing, China). Animals were housed in an SPF‐grade environment. All experiments were performed in accordance with the guidelines of the Institutional Animal Care and Use Committee. The experimental protocol was also approved by the Laboratory Animal Ethics Committee of Central South University (Ethics No. 202411225). Cell culture was performed before the experiment, and at the time of the experiment, cells were digested with 0.25% trypsin, centrifuged, resuspended in precooled serum‐free RPMI 1640 medium, adjusted to a concentration of 1 × 10^7^ cells/mL, and injected with 100 μL of T24 cell suspension in the unilateral groin of each nude mouse. The animals were randomly divided into three groups of 6 animals each: the control group, the low‐concentration group, and the high‐concentration group. From the third day after inoculation, the animals were observed every 2 days, and the intraperitoneal drug injection was started at the 10th day. The control group was injected with DMSO. The concentration of the drug was 25 mg/kg in the low‐concentration group and 50 mg/kg in the high‐concentration group for 14 consecutive days. At the end of the day, the animals were euthanized, and the subcutaneous tumors were removed for photographing and weighing.

### 2.5. Transmission Electron Microscopy Ultrastructure Observation

Fresh tissue was initially fixed with electron microscope fixative (Servicebio, China) and then transferred to a 1% osmium tetroxide solution for secondary fixation. The samples were rinsed with phosphate buffer and dehydrated through a graded alcohol series. Acetone mixed with 812 embedding resin was used for infiltration and embedding. The resin‐embedded blocks were sectioned at 1.5 *μ*m thickness, stained with toluidine blue, and observed under a microscope for positioning. Next, ultrathin sections (60–80 nm) were cut from the resin block and stained with 2% uranyl acetate followed by 2.6% lead citrate. After drying, the sections were examined with a transmission electron microscope (JEM1400PLUS, Japan) to observe mitochondrial structure and morphology.

### 2.6. Hematoxylin–Eosin Stain (HE Stain)

Freshly taken organ tissues were fixed in 10% neutral formalin, dehydrated with gradient ethanol, then embedded in paraffin, cut into sections of 5~7 *μ*m thickness, and adhered to slides. After deparaffinization, the nuclei of the cells were dipped and stained with hematoxylin staining solution, the cytoplasm of the cells was re‐stained with eosin staining solution, and the slices were sealed with neutral tree resin. The images were observed and captured under the microscope.

### 2.7. Quantitative Real‐Time PCR (qRT‐PCR)

Total cellular RNA was extracted using a TRIzol kit (TransGen Biotech, China) after collection of cellular precipitates, and cDNA was obtained by reverse transcription using HiScript II Q RT SuperMix for qPCR (+gDNA Wiper) kit (Vazyme, China). qRT‐PCR experiments were performed using a ChamQ Universal SYBR qPCR Premix from Vazyme in 96‐well reaction plates with a Bio‐Rad Real‐Time Polymerase Chain Reaction System. Gene expression levels were assessed by RQ = 2^(−)(*Δ* *Δ*Ct)^, and three replicate wells were designed for all experiments. The primers used in qRT‐PCR are listed below:

GAPDH‐F: GTCTCCTCTGACTTCAACAGCG,

GAPDH‐R: ACCACCCTGTTGCTGTAGCCAA;

FOXO4‐F: ACGAGTGGATGGTCCGTACTGT,

FOXO4‐R: CCTTGATGAACTTGCTGTGTGCAGG;

BCL6‐F: CATGCAGAGATGTGCCTCCACA,

BCL6‐R: TCAGAGAAGCGGCAGTCACACT;

Bcl‐xL‐F: GCCACTTACCTGAATGACCACC,

Bcl‐xL‐R: AACCAGCGGTTGAAGCGTTCCT;

Caspase‐9‐F: GTTTGAGGACCTTCGACCAGCT,

Caspase‐9‐R: CAACGTACCAGGAGCCACTCTT;

Caspase‐3‐F: GGAAGCGAATCAATGGACTCTG,

Caspase‐3‐R: GCATCGACATCTGTACCAGACC.

### 2.8. Western Blot

Cell precipitates were collected and lysed by adding mixed lysis solution (RIPA : PMSF = 100 : 1), and the supernatant was collected after low‐temperature and high‐speed centrifugation. The protein concentration was determined by using a BCA assay kit (NCM Biotech, China). Protein samples were separated by SDS‐PAGE, transferred to PVDF membranes (ThermoFisher Scientific, China), 5% BSA solution (closed and incubated overnight at 4°C with the corresponding antibodies). The following day, the corresponding secondary antibodies were incubated for 2 h on a shaker at room temperature. The immunoreactive bands were detected using the enhanced chemiluminescence detection reagent (ThermoFisher Scientific, China). Results were analyzed using ImageJ software. The antibodies used for Western blot were as follows: GAPDH (1:8000, #AC002, ABClonal), *β*‐actin (1:8000, #AC004, ABClonal), FOXO4 (1:500, #AC3307, ABClonal), BCL6 (1:500, #AC7173, ABClonal), Bcl‐xL (1:500, #AC0209, ABClonal), Caspase‐9 (1:500, #AC2636, ABClonal), and Caspase‐3 (1:500, #AC11319, ABClonal).

### 2.9. Flow Cytometry

Using an Annexin V‐FITC/PI double staining apoptosis detection kit (Solarbio, China), cells were collected by centrifugation and resuspended in 100 *μ*L binding buffer. Annexin V‐FITC fluorescent probe was added and then incubated with PI staining solution under low light conditions. The assay was performed by flow cytometry, the excitation light was 488 nm, and FITC (green fluorescence, to detect Annexin V) and PI (red fluorescence, to detect necrotic cells/late apoptotic cells) were collected separately. The assay was performed by flow cytometry, and the excitation light was selected at 488 nm. The signals of FITC (green fluorescence, detecting Annexin V) and PI (red fluorescence, detecting necrotic/late apoptotic cells) channels were collected separately, and the data of at least 20,000 cells were obtained. Scatter plots were drawn using FlowJo software to divide four quadrants: the lower left quadrant for live cells (Annexin V^−^/PI^−^), the lower right quadrant for early apoptotic cells (Annexin V^+^/PI^−^), the upper left quadrant for PI single‐positive cells (Annexin V^−^/PI^+^), and the upper right quadrant for late apoptotic/necrotic cells (Annexin V^+^/PI^+^), and the proportion of apoptotic cells was calculated.

### 2.10. Mitochondrial Membrane Potential (*Δ* *Ψ* *m*) Detection

The JC‐1 assay was performed using the mitochondrial membrane potential assay kit with JC‐1 (Beyotime, China). Cells were collected, centrifuged, and resuspended (cell concentration of 1 × 10^6^ cells/mL). JC‐1 storage solution was added and incubated to avoid light, and then, staining buffer was added to detect the assay using flow cytometry. The excitation light was selected to be 488 nm. The signals of the FL1 channel (525 nm, green fluorescence of JC‐1 monomer) and the FL2 channel (590 nm, red fluorescence of JC‐1 polymer) were collected, and data of at least 20,000 cells were acquired for each sample. The ratio of red fluorescence to green fluorescence intensity (*R*/*G* value) was calculated by flow analysis software.

### 2.11. Statistical Analysis

All experiments were carried out in three or more independent replicates with at least three replicate wells per experiment. Results are expressed as mean (Mean) ± standard deviation (SD). All data analyses and graphing were performed using GraphPad Prism 8.0 and the built‐in data analysis. For paired organization data analysis, the “paired *t*‐test” was used for statistical analysis, and the “unpaired two‐tailed *t*‐test” was used for analysis of differences between groups. *p* < 0.05 indicates statistical difference,  ^∗^ *p* < 0.05,  ^∗∗^ *p* < 0.01,  ^∗∗∗^ *p* < 0.001, and  ^∗∗∗∗^ *p* < 0.0001.

## 3. Results

### 3.1. FLLL31 Inhibits Malignant Biological Behavior of BC Cells

We used the Natural Product Library (Selleck China) to screen potential therapeutic drugs for BC, and FLLL31 attracted our attention after treatment of BC cells with a gradient concentration of the drug. FLLL31 (C25H28O6) (Figure 1a) is a diketone analog of curcumin. Some studies have reported that FLLL31 can be used to alleviate rhinitis [27], induce apoptosis in pancreatic and breast cancer cells [5], and so on. After different concentrations of FLLL31 were treated for 24 h, we determined the drug treatment concentration by measuring the IC_50_ through the CCK‐8 assay (Figure 1b), and 2.5 and 5 *μ*M were used as the low‐ and high‐concentration treatment groups of FLLL31, respectively. This concentration setting was used in all subsequent experiments. The following experiments were conducted at these concentrations. To further investigate the effects of FLLL31 on the biological functions of BC cells, we performed a series of in vitro experiments to evaluate the effects of FLLL31 on the proliferation, invasion, and migration of BC cells after treating the cells with different concentrations of FLLL31. We could clearly demonstrate the effects of FLLL31 on the proliferation, invasion, and migration of BC cells by using the CCK‐8 assay (Figure 1c), the clone formation assay (Figure 1d), and the EdU assay (Figure 1e). We can clearly see that FLLL31 can significantly inhibit the proliferation of BC cells, and the inhibitory effect increases with the concentration of the drug treatment. Similarly, the Transwell assay also showed that FLLL31 can significantly inhibit the migration (Figure 1f) and invasion (Figure 1g) of BC cells, and the inhibitory effect is dose‐dependent with the increasing concentration of the drug treatment. The inhibitory effect was enhanced in a dose‐dependent manner with the increase in drug concentration.

Figure 1FLLL31 suppresses malignant phenotypes in bladder cancer cells. (a) Chemical structure of FLLL31 (C_25_H_28_O_6_). (b) Dose–response curves and IC_50_ values of FLLL31 in T24/5637 cells (CCK‐8 assay, 24 h). (c) Proliferation inhibition by FLLL31 (CCK‐8 assay; *p* < 0.01 vs. DMSO). (d) FLLL31 treatment dose‐dependently reduced colony formation capacity in bladder cancer cells (crystal violet staining). (e) FLLL31 treatment dose‐dependently attenuated DNA replication in bladder cancer cells (EdU assay AF488 fluorescence). (f) FLLL31 treatment dose‐dependently suppressed cell migration (Transwell assay). (g) FLLL31 treatment dose‐dependently impaired Matrigel invasion (Matrigel‐coated Transwell assay). FLLL31 was used at a concentration of 2.5/5 *μ*M. The treatment duration was 24 h. Mean ± SD; *n* = 3;  ^∗^ *p* < 0.05,  ^∗∗^ *p* < 0.01,  ^∗∗∗^ *p* < 0.001, and  ^∗∗∗∗^ *p* < 0.0001.

**Figure:**
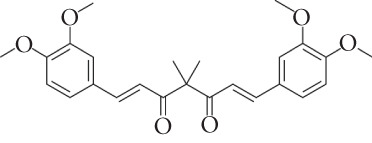
(a)

**Figure:**
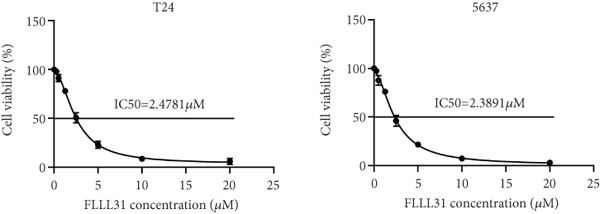
(b)

**Figure:**
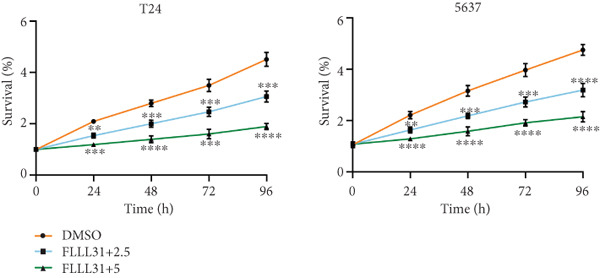
(c)

**Figure:**
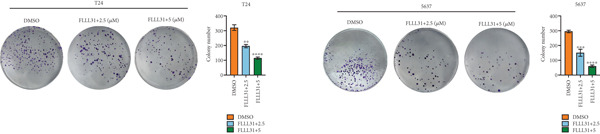
(d)

**Figure:**
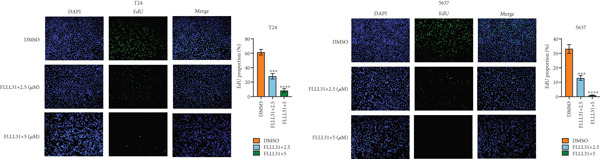
(e)

**Figure:**
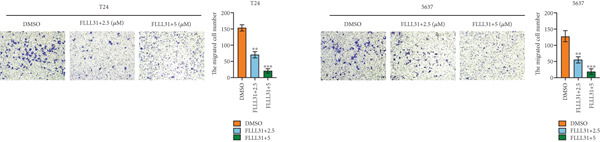
(f)

**Figure:**
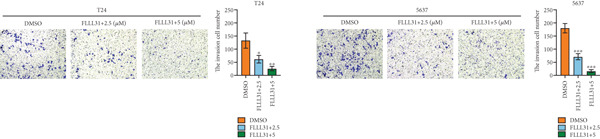
(g)

### 3.2. FLLL31 Promotes Apoptosis in BC Cells Through the FOXO4 Signaling Pathway

In order to further investigate the molecular mechanism of FLLL31 inhibiting the malignant biological phenotype of BC, we selected T24 cells for RNA‐sequencing (RNA‐seq) analysis. The DMSO‐treated group served as the negative control, and the group treated with 2.5 *μ*M FLLL31 was used as the experimental group. RNA‐seq results revealed 413 upregulated and 186 downregulated genes following FLLL31 treatment (Figure 2a). Among these, FOXO4 and BCL6 expressions were significantly elevated in the FLLL31‐treated group compared to the control. Integration of the TF target Sankey analysis (Figure 2b) and existing literature suggested that FLLL31 may promote FOXO4 expression, leading to subsequent upregulation of BCL6. After we treated T24 and 5637 with different concentrations of FLLL31 for 24 h, we observed the changes in cell morphology by electron microscopy, which showed that the mitochondria of cells treated with low concentration of FLLL31 were balloon‐like, the cristae were blurred, and the number of cristae was reduced, and the chromatin coalesced into clumps and aggregated toward the edge, the outer mitochondrial membranes of some mitochondria ruptured, and the endoplasmic reticulum dilated into a vesicle‐like structure after the treatment of cells with high concentration of FLLL31 (Figure 2c), which showed the obvious apoptotic morphology. Then, we treated the cells in the same way to observe the changes in cell morphology. After treating logarithmic stage BC cells with different concentrations of FLLL31, we showed a dose‐dependent increase in early apoptosis (Q3, Annexin V^+^/PI^−^) by Annexin V‐FITC/PI double‐staining flow cytometry (Figure 2d). In apoptosis, mitochondria are the key hub, and we further verified the results by mitochondrial membrane potential assay. JC‐1 results showed that the red/green fluorescence intensity ratio (*R*/*G* ratio) decreased significantly after FLLL31 treatment in BC cells, and the trend of the decrease increased with the concentration of the drug treatment in a dependent manner (Figure 2e). The above results suggested that FLLL31 could exert its biological effects by inducing apoptosis in BC cells. Therefore, in combination with the RNA‐seq results, we believe that FLLL31 plays a role in the apoptotic function of BC cells through the upregulation of BCL6 by promoting the expression of FOXO4 and the upregulation of BCL6. It has been demonstrated that when BCL6 is upregulated, apoptosis can be induced by inhibiting the antiapoptotic factor Bcl‐xL [28, 29].

Figure 2FLLL31 induces mitochondrial apoptosis via FOXO4/BCL6 axis activation. (a) RNA‐seq volcano plot: differentially expressed genes in FLLL31‐treated T24 cells (2.5 *μ*M, 24 h; |log2FC| > 1, FDR < 0.05). (b) TF‐target network: FOXO4‐BCL6 regulatory linkage (Sankey diagram). (c) Electron microscope image of bladder cancer cells after FLLL31 treatment (scale bar: 5, 2 *μ*m). (d) Flow cytometry showed that FLLL31 induced apoptosis in bladder cancer cells (Annexin V^+^/PI^−^; flow cytometry; FLLL31 2.5/5 *μ*M, 24 h). (e) The JC‐1 results indicated that FLLL31 downregulated the *R*/*G* ratio of bladder cancer cells (FLLL31 2.5/5 *μ*M, 24 h). (f–j) Dose‐dependent mRNA upregulation of FOXO4, BCL6, Caspase‐9, and Caspase‐3 and downregulation of Bcl‐xL (qRT‐PCR; FLLL31 2.5/5 *μ*M, 24 h). (k–l) Dose‐dependent protein expression upregulation of FOXO4, BCL6, Caspase‐9, and Caspase‐3 and downregulation of Bcl‐xL (Western blot; FLLL31 2.5/5 *μ*M, 24 h). FLLL31 was used at a concentration of 2.5/5 *μ*M. The treatment duration was 24 h. Mean ± SD; *n* = 3;  ^∗^ *p* < 0.05,  ^∗∗^ *p* < 0.01,  ^∗∗∗^ *p* < 0.001, and  ^∗∗∗∗^ *p* < 0.0001.

**Figure:**
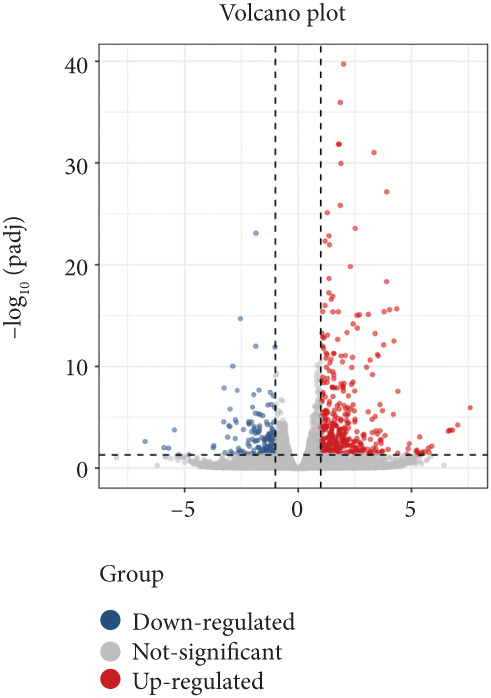
(a)

**Figure:**
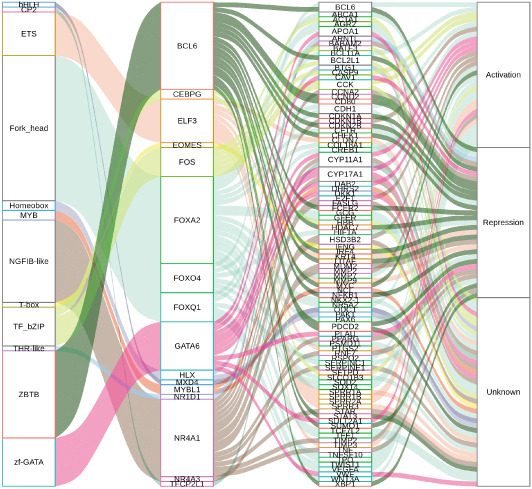
(b)

**Figure:**
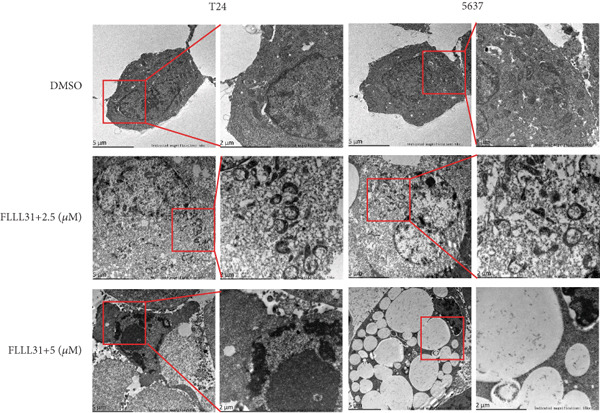
(c)

**Figure:**
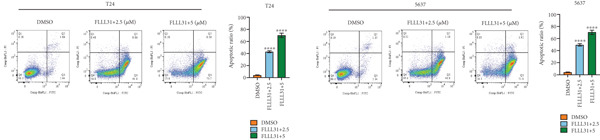
(d)

**Figure:**
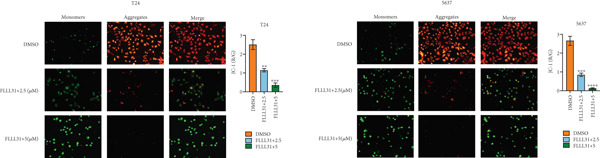
(e)

**Figure:**
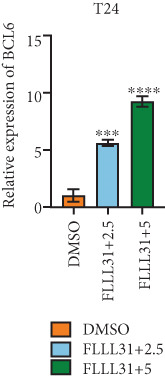
(f)

**Figure:**
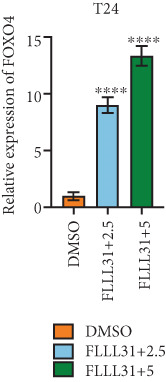
(g)

**Figure:**
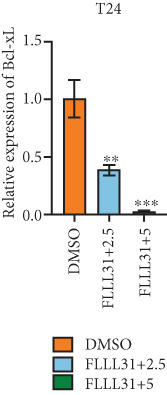
(h)

**Figure:**
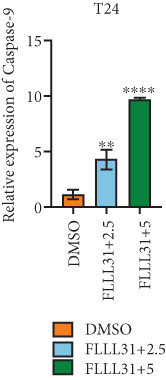
(i)

**Figure:**
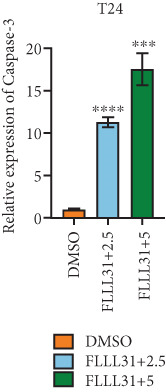
(j)

**Figure:**
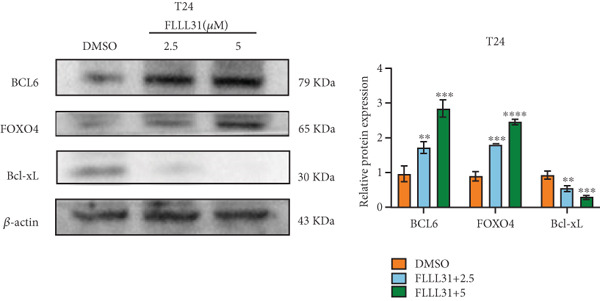
(k)

**Figure:**
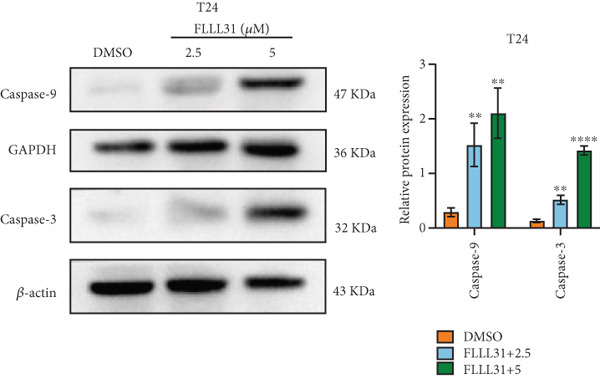
(l)

Given that Bcl‐xL is a signature molecule of mitochondrial apoptosis [30], we propose that FLLL31 triggers mitochondria‐dependent apoptosis in BC cells via a FOXO4‐mediated upregulation of BCL6, culminating in the repression of the antiapoptotic factor Bcl‐xL. We investigated this underlying molecular mechanism using qRT‐PCR and Western blot analyses. FLLL31 treatment induced concentration‐dependent upregulation of FOXO4, BCL6, Caspase‐9, and Caspase‐3 at both mRNA (Figures 2f, 2g, 2i, and 2j) and protein (Figure 2k,l) levels. Conversely, it downregulated Bcl‐xL expression in a dose‐dependent manner (Figure 2h,k), supporting our hypothesis.

These results support a model in which FLLL31 enhances FOXO4 expression, leading to BCL6 upregulation. BCL6 then represses Bcl‐xL, increasing MMP and releasing proapoptotic factors that activate Caspase‐9 and Caspase‐3. This cascade ultimately induces apoptosis and inhibits BC progression.

### 3.3. In Vivo Experiments Demonstrate That FLLL31 Significantly Inhibits BC Growth

At the cellular level, we can see that FLLL31 significantly inhibited the malignant phenotype of BC cells, and then, we will further verify the above experimental findings at the in vivo level. We selected BC cells T24 to construct a BC‐subcutaneous tumor mouse model, and we proposed 25 mg/kg/day as the low concentration injection and 50 mg/kg/day as the high concentration injection. We injected the drug intraperitoneally every day and then euthanized the mice for 14 days (Figure 3a). The results showed that after injection of FLLL31, mice′s subcutaneous tumor size (Figure 3b), volume (Figure 3c), and weight (Figure 3d) were significantly reduced, the tumor growth rate (Figure 3e) was obviously slowed down, and the inhibitory effect was enhanced in a concentration‐dependent manner, suggesting that FLLL31 could effectively inhibit the growth of BC cells. Additionally, histopathological examination of major organs (heart, liver, spleen, lungs, and kidneys) via HE staining (Figure 3f) revealed no significant structural abnormalities or pathological alterations in either the dimethyl sulfoxide (DMSO) control group or the drug‐treated cohort. These findings collectively demonstrate that FLLL31 exhibits no apparent toxicity toward vital organs within the experimental dosage range, indicating its favorable safety profile.

Figure 3FLLL31 inhibits bladder cancer growth in vivo with minimal toxicity. (a) Experimental timeline: T24 xenograft establishment, FLLL31 dosing (FLLL31 25/50 mg/kg/day, ip, 14 days), and endpoint analysis. (b) Photo of subcutaneous tumors of bladder cancer. (c) Volumes of subcutaneous tumors in bladder cancer. (d) Weights of subcutaneous tumors in bladder cancer. (e) Tumor growth inhibition rates. (f) HE‐stained vital organs (heart, liver, spleen, lung, and kidney) showing no pathological alterations (scale bar: 50 *μ*m). Mean ± SD; *n* = 6;  ^∗^ *p* < 0.05,  ^∗∗^ *p* < 0.01,  ^∗∗∗^ *p* < 0.001, and  ^∗∗∗∗^ *p* < 0.0001. The experimental protocol was also approved by the Laboratory Animal Ethics Committee of Central South University (Ethics No. 202411225).

**Figure:**
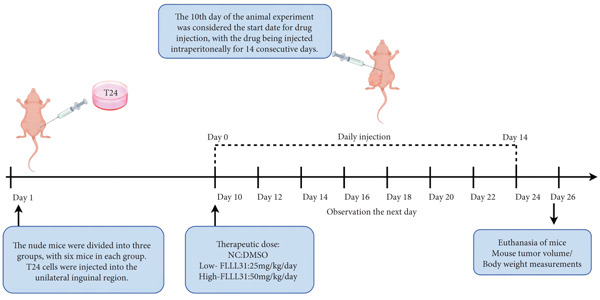
(a)

**Figure:**
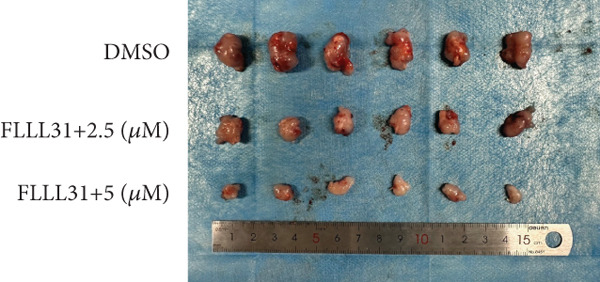
(b)

**Figure:**
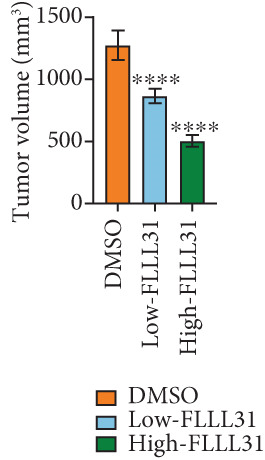
(c)

**Figure:**
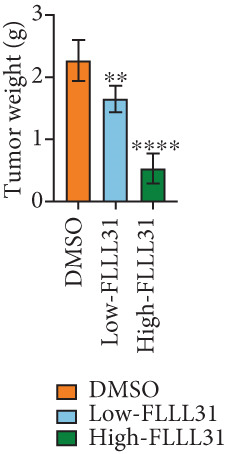
(d)

**Figure:**
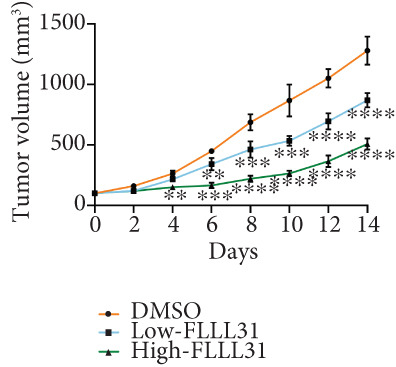
(e)

**Figure:**
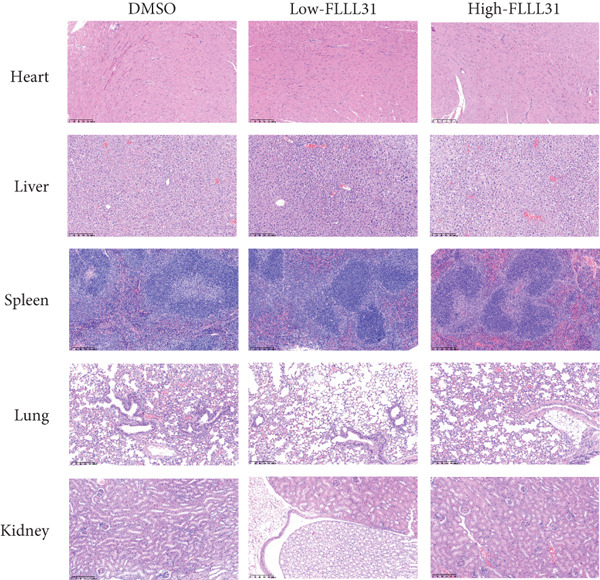
(f)

### 3.4. FOXO4 Acts as an Upstream Regulator to Mediate FLLL31‐Induced Apoptosis in BC Cells by Upregulating BCL6

Previous studies indicated that FLLL31 activates the apoptotic signaling pathway by upregulating FOXO4 and BCL6 expressions. To determine the necessity of FOXO4 and BCL6 in FLLL31‐induced apoptosis of BC cells, we employed lentivirus‐mediated knockdown of each gene individually and assessed whether FLLL31′s regulation of apoptosis‐related genes depends on FOXO4 and BCL6.

Knockdown of BCL6 significantly reduced its mRNA and protein levels (Figure 4a,g). Treatment of T24‐shBCL6 cells with increasing concentrations of FLLL31, followed by qRT‐PCR and Western blot analysis, revealed that BCL6 knockdown markedly attenuated FLLL31′s effects on the expression of BCL6 (Figure 4b,h), Bcl‐xL (Figure 4d,h), Caspase‐9 (Figure 4e,i), and Caspase‐3 (Figure 4f,i). In contrast, changes in FOXO4 mRNA (Figure 4b) and protein (Figure 4h) levels induced by FLLL31 in the BCL6‐knockdown group largely remained consistent with those in the control group (Figure 2g,k). Similarly, FOXO4 knockdown effectively reduced FOXO4 mRNA (Figure 4j) and protein (Figure 4p) expressions. Furthermore, FOXO4 depletion significantly diminished FLLL31′s impact on the mRNA and protein levels of BCL6 (Figure 4k,q), FOXO4 (Figure 4l,q), Bcl‐xL (Figure 4m,q), Caspase‐9 (Figure 4n,r), and Caspase‐3 (Figure 4o,r).

Figure 4FOXO4/BCL6 knockdown abrogates FLLL31‐induced apoptotic signaling. (a) Lentiviral knockdown of BCL6 resulted in significantly lower mRNA levels of BCL6 compared to the nonknockdown group. (b–f) BCL6 knockdown was performed, followed by qRT‐PCR to assess FLLL31‐mediated changes in the mRNA expression of (b) BCL6, (c) FOXO4, (d) Bcl‐xL, (e) Caspase‐9, and (f) Caspase‐3. (g) Lentiviral knockdown of BCL6 resulted in significantly lower protein levels of BCL6 compared to the nonknockdown group. (h, i) Knockdown of BCL6 and Western blot analysis were used to detect the effects of FLLL31 on the protein levels of BCL6, FOXO4, (f) Bcl‐xL, Caspase‐9, and (g) Caspase‐3. (j) Lentiviral knockdown of FOXO4 resulted in significantly lower mRNA levels of FOXO4 compared to the nonknockdown group. (k–o) FOXO4 knockdown was performed followed by qRT‐PCR to assess FLLL31‐mediated changes in the mRNA expression of (k) BCL6, (l) FOXO4, (m) Bcl‐xL, (n) Caspase‐9, and (o) Caspase‐3. (p) Lentiviral knockdown of FOXO4 resulted in significantly lower protein level of FOXO4 compared to the nonknockdown group. (q, r) Knockdown of FOXO4 and Western blot analysis were used to detect the effects of FLLL31 on the protein levels of BCL6, FOXO4, (q) Bcl‐xL, Caspase‐9, and (r) Caspase‐3. FLLL31 was used at a concentration of 2.5/5 *μ*M. The treatment duration was 24 h. Mean ± SD; *n* = 3;  ^∗^ *p* < 0.05,  ^∗∗^ *p* < 0.01,  ^∗∗∗^ *p* < 0.001, and  ^∗∗∗∗^ *p* < 0.0001.

**Figure:**
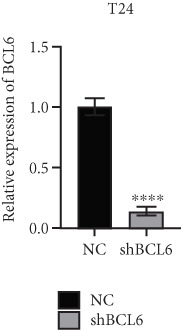
(a)

**Figure:**
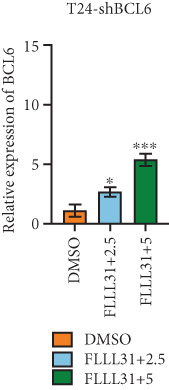
(b)

**Figure:**
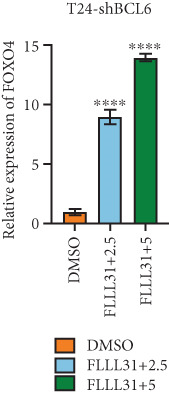
(c)

**Figure:**
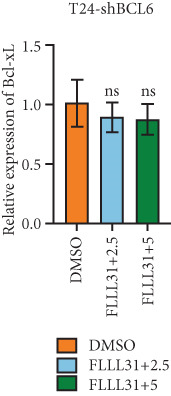
(d)

**Figure:**
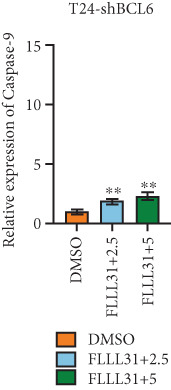
(e)

**Figure:**
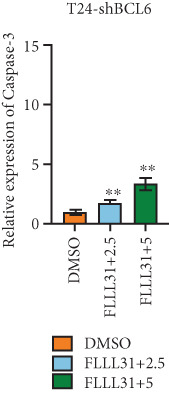
(f)

**Figure:**
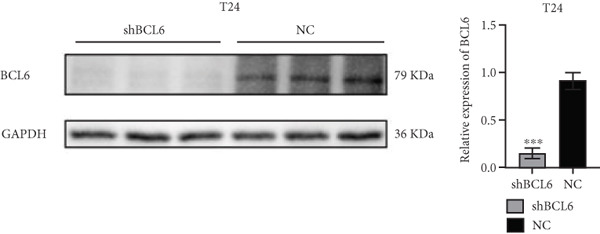
(g)

**Figure:**
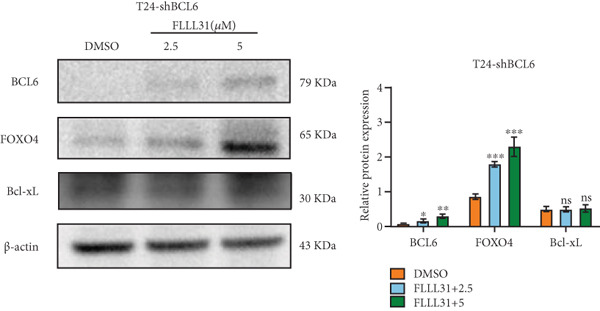
(h)

**Figure:**
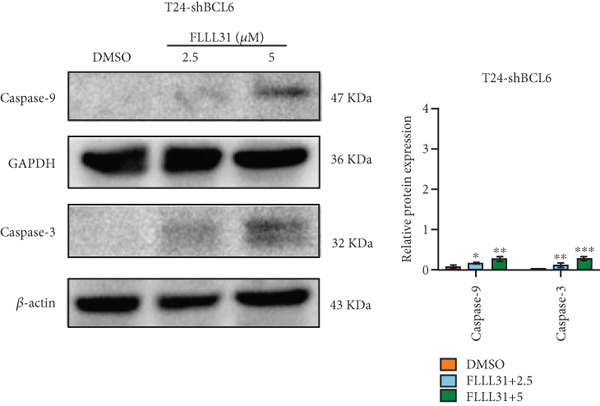
(i)

**Figure:**
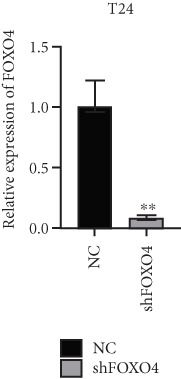
(j)

**Figure:**
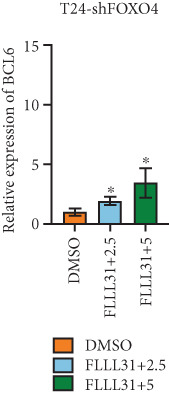
(k)

**Figure:**
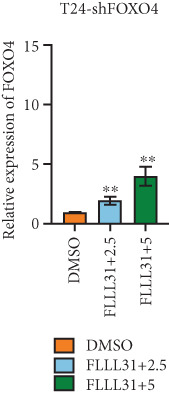
(l)

**Figure:**
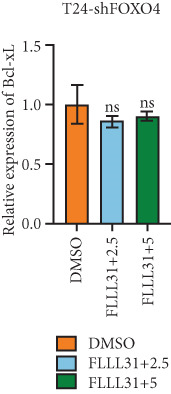
(m)

**Figure:**
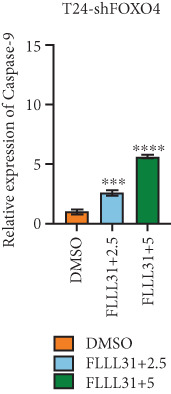
(n)

**Figure:**
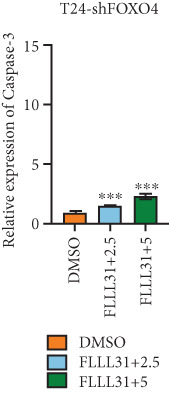
(o)

**Figure:**
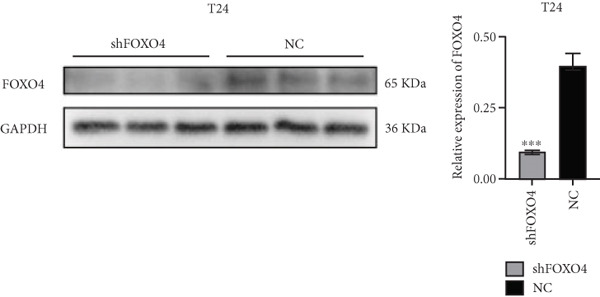
(p)

**Figure:**
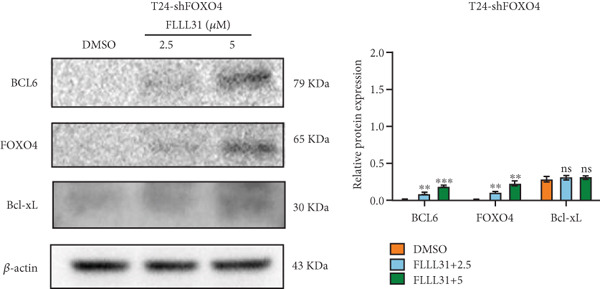
(q)

**Figure:**
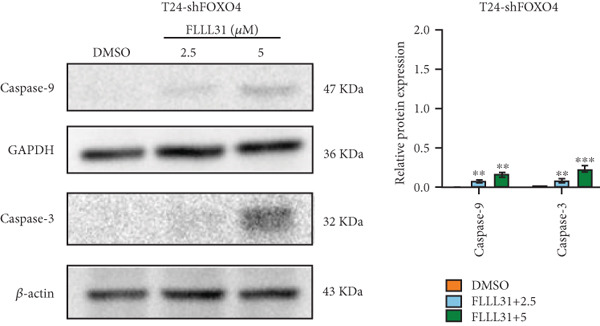
(r)

The results indicate that FLLL31 can affect the expression level of BCL6 by altering the expression of FOXO4, thereby activating the mitochondria‐dependent apoptotic pathway.

### 3.5. Knockdown of BCL6 and FOXO4 Inhibits Apoptosis Induction and Malignant Phenotype Modulation by FLLL31 in BC Cells

Previous findings have confirmed that during FLLL31‐induced apoptosis in BC cells, FOXO4 transcriptionally regulates BCL6 expression and activates the mitochondria‐dependent apoptotic pathway, collectively establishing a hierarchically organized signaling axis among these components. In order to further verify the functional significance of FOXO4 and BCL6 in the regulation of malignant phenotypes of BC cells, we used lentivirus to knock down FOXO4 and BCL6 and then verified their regulatory mechanisms with the help of a series of biological function experiments. Knocking down either FOXO4 or BCL6 significantly attenuated FLLL31′s inhibitory effects on T24 cell proliferation (Figures 5a, 5b, and 5c), invasion (Figure 5d), and migration (Figure 5e). At 2.5 *μ*M FLLL31, phenotypic inhibition was minimal in knockdown cells compared to controls; at 5 *μ*M, inhibition was observed but remained significantly weaker than in nonknockdown cells treated equivalently.

Figure 5Depletion of BCL6 or FOXO4 abrogates FLLL31‐induced apoptosis and malignancy in bladder cancer cells. (a–c) The effects of FLLL31 on the proliferation of bladder cancer cells after knockdown of BCL6 or FOXO4 were detected through (a) colony formation assay, (b) CCK‐8, and (c) EdU assays. (d) The effect of FLLL31 on the migration of bladder cancer cells after knockdown of BCL6 or FOXO4 was detected through the Transwell assay. (e) The effect of FLLL31 on the invasion of bladder cancer cells after knockdown of BCL6 or FOXO4 was detected through the Matrigel‐coated Transwell assay. (f–h) The effects of FLLL31 on the apoptosis of bladder cancer cells after knockdown of BCL6 or FOXO4 were detected by (f) electron microscopy, (g) flow cytometry, and (h) JC‐1. Mean ± SD; *n* = 3;  ^∗^ *p* < 0.05,  ^∗∗^ *p* < 0.01,  ^∗∗∗^ *p* < 0.001, and  ^∗∗∗∗^ *p* < 0.0001.

**Figure:**
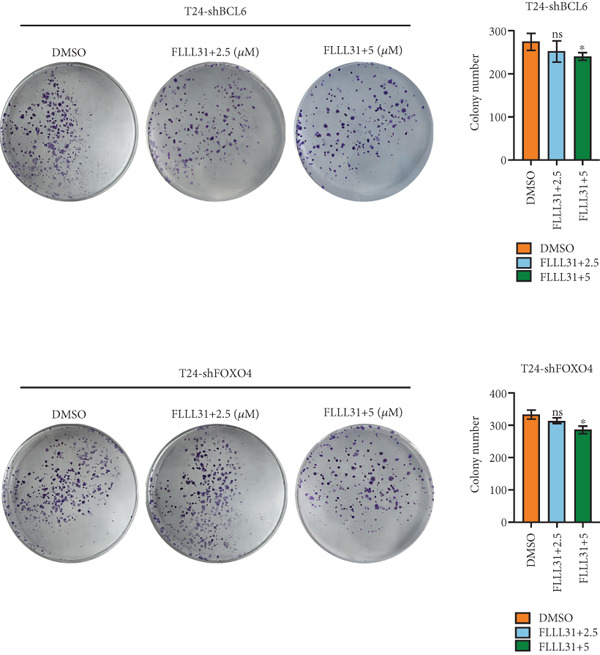
(a)

**Figure:**
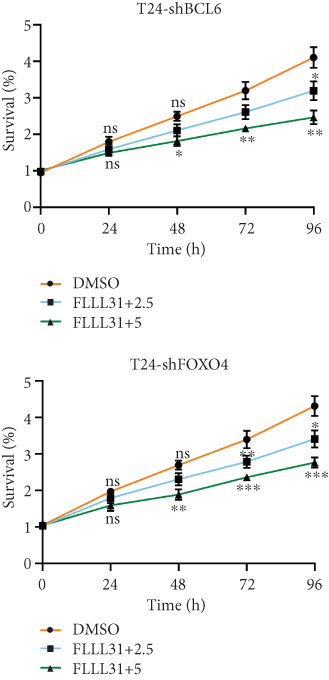
(b)

**Figure:**
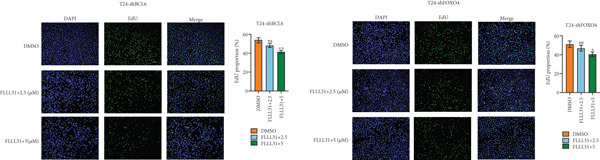
(c)

**Figure:**
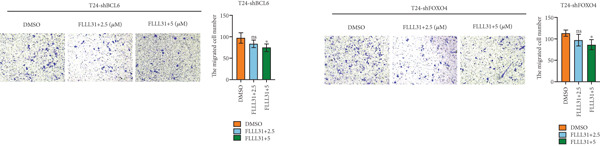
(d)

**Figure:**
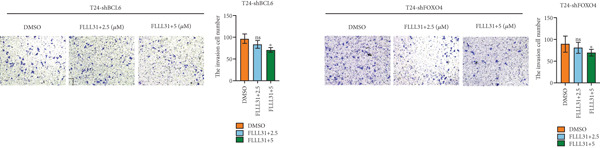
(e)

**Figure:**
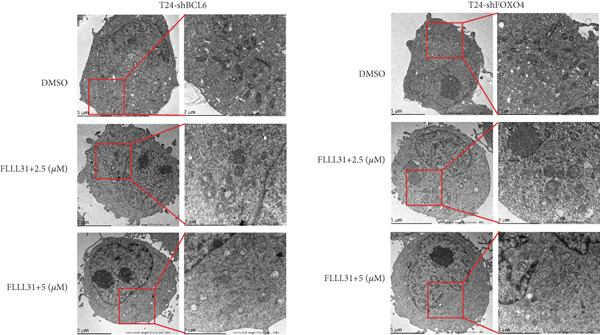
(f)

**Figure:**
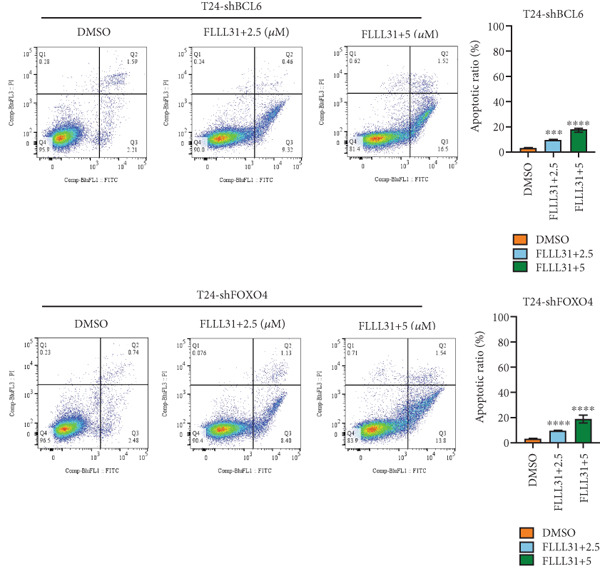
(g)

**Figure:**
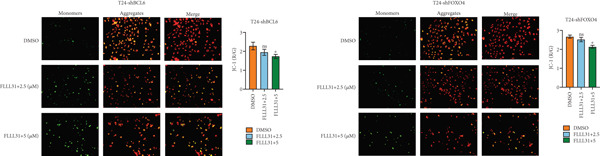
(h)

Consistent with FLLL31′s reliance on FOXO4/BCL6 for apoptosis induction, electron microscopy revealed only mild mitochondrial swelling without characteristic apoptotic ultrastructure in knockdown cells treated with FLLL31 (Figure 5f). Flow cytometry confirmed drastically reduced total apoptosis rates in knockdown cells versus wild‐type cells at equivalent concentrations (Figure 5g). Similarly, the decrease in mitochondrial membrane potential (*Δ* *Ψ* *m*, measured by *R*/*G* ratio) was insignificant at 2.5 *μ*M FLLL31 and markedly weaker at 5 *μ*M in knockdown cells compared to controls (Figure 5h).

## 4. Discussion

Despite advancements in BC therapy, clinical outcomes remain suboptimal, underscoring the critical need for novel therapeutic agents and targets. Through screening of compound libraries, we identified FLLL31, a synthetic diketone analog of curcumin, as a potent inhibitor of BC cell growth—a finding previously unreported. Notably, FLLL31 exhibits enhanced chemical stability and reduced cytotoxicity toward normal cells compared to its parent compound curcumin [31]. Our mechanistic investigations demonstrate that FLLL31 suppresses malignant progression in BC by inducing apoptosis via modulation of the FOXO4/BCL6 signaling axis.

Our in vitro analyses established that FLLL31 significantly suppresses proliferation, migration, and invasion capacities in BC cells. To delineate the mechanistic basis, we conducted RNA‐seq followed by integrative bioinformatic interrogation. Critically, the combined examination of RNA‐seq data and TF target Sankey network diagrams revealed conspicuous upregulation of FOXO4 and BCL6.

FOXO4, a core member of the FOXO TF family, governs pivotal cellular processes including metabolic adaptation, cell cycle arrest, apoptotic execution, and homeostatic maintenance via transcriptional modulation of downstream effectors [32–34]. Some studies have pointed out that FOXO4 activates apoptosis by induction of the BCL6 transcriptional repressor [35]. Apoptosis, a genetically programmed and self‐driven cellular demise process triggered under specific physiological or pathological conditions [36], plays pivotal roles in embryogenesis, tissue homeostasis maintenance, and immune regulation [37]. Its dysregulation is intricately linked to cancer pathogenesis, thereby constituting a critical target for therapeutic strategies including targeted therapy and immunotherapy [38, 39]. Evidence indicates that elevated FOXO4 expression markedly suppresses glycolytic activity in gastric cancer (GC) cells [40]. Similarly, in colorectal cancer (CRC), FOXO4 overexpression curtails cell migration and metastasis through reinforcement of the APC2/*β*‐catenin signaling axis [41]. Taken together with the current study, these findings support the notion that FOXO4 may influence tumor proliferation, migration, and invasion via multiple mechanisms, including metabolic reprogramming, activation of tumor‐suppressive pathways, or direct induction of cancer cell apoptosis.

Originally identified as a proto‐oncogene in B‐cell lymphomas, BCL6 drives malignant phenotypes by repressing proliferation and DNA damage checkpoints, as well as by blocking terminal B‐cell differentiation [42]. The biological functions of BCL6, however, vary across cell types. For instance, in lung cancer, BCL6 is regulated by the MAPK/ELK1 axis and facilitates KRAS‐driven tumorigenesis [43]. In germinal center B cells, BCL6 promotes apoptosis through repression of BCL2 [44]. This protein exerts a dichotomous effect on apoptotic regulation: It suppresses p53‐mediated apoptosis via transcriptional repression of p53 [45] yet promotes intrinsic apoptosis by inhibiting Bcl‐xL expression [35]. Our experimental data demonstrate that escalating concentrations of FLLL31 induce dose‐dependent upregulation of FOXO4 and BCL6, concomitant with suppression of Bcl‐xL expression. These findings collectively suggest that FLLL31 may impede BC progression by triggering the intrinsic apoptotic pathway. This mechanistic hypothesis is further substantiated by the observed activation of Caspase‐9 and Caspase‐3 upon Bcl‐xL downregulation.

Lentivirus‐mediated knockdown experiments revealed that decreased expression of BCL6 did not alter FOXO4 expression levels yet significantly attenuated FLLL31‐induced apoptosis. Conversely, FOXO4 silencing concurrently reduced both BCL6 expression and apoptotic activation. These data definitively establish that FLLL31 exerts its antitumor effects in BC primarily through the FOXO4/BCL6 axis–mediated intrinsic apoptotic pathway.

The novelty of this study lies in the first demonstration that the natural product‐derived molecule FLLL31 induces tumor cell apoptosis by targeting the FOXO4/BCL6 axis. This delineates a novel mechanism of action and provides a potential precision therapeutic strategy for BC distinct from conventional chemotherapy. This mechanism provides a distinct therapeutic strategy for BC that diverges fundamentally from conventional chemotherapy. Critically, FLLL31 exhibits enhanced bioavailability and metabolic stability relative to parental curcumin, enabling selective tumor cytotoxicity via coordinated upregulation of FOXO4, activation of BCL6, and suppression of Bcl‐xL‐mediated mitochondrial apoptotic inhibition. Such target specificity aligns with contemporary precision oncology principles. Furthermore, in vivo validation confirmed potent dose‐dependent suppression of BC xenografts without inducing significant histopathological alterations in vital organs, indicating a favorable preclinical safety profile.

The rational use of integrative machine learning models can significantly improve the probability of early detection of cancer [46], and in future studies, we will further explore whether FOXO4 can be one of the key markers for the diagnosis and treatment of BC based on the current results.

## 5. Conclusion

In the present study, we found that FLLL31 activates the mitochondria‐dependent apoptosis pathway by targeting the FOXO4/BCL6 axis to inhibit the malignant phenotype of BC cells, which provides a new drug candidate and target for the treatment of BC. The hierarchical relationship and molecular regulatory mechanism of the signaling pathway revealed in this study not only expand the research on the mechanism of FLLL31 in urological tumors but also have great significance for the development of precise targeted therapeutic strategies based on natural drug derivatives. In the future, we can further focus on the upstream target of FLLL31, long‐term drug safety, and clinical biomarker screening, so as to promote the translation of FLLL31 from basic research to clinical application.

## Ethics Statement

The experimental protocol was also approved by the Laboratory Animal Ethics Committee of Central South University (Ethics No. 202411225).

## Disclosure

The authors approved the submitted version.

## Conflicts of Interest

The authors declare no conflicts of interest.

## Author Contributions

Yu Han and Zhengyan Tang conceived and designed the experiments. Yu Han and Jingxuan Peng performed the experiments and did data analysis. Yu Han, Wenjie Yan, and Ziqiang Wu provided technical support and critical comments. Yu Han drafted the manuscript. Zhengyan Tang provided funding support. All authors participated in the revision of the manuscript.

## Funding

This work was supported by the Natural Science Foundation of China, No. 81700667.

## Supporting information


**Supporting Information** Additional supporting information can be found online in the Supporting Information section. This Excel file contains the raw experimental data used to generate all figures and statistical analyses presented in this manuscript. The data are organized into separate sheets corresponding to Figures 1b, 1c, and 1d.

## Data Availability

The authors confirm that all raw data generated in this study have been provided as a supporting information data file (Source data.xlsx).

## References

[1] Huang P. , Wang J. , Yu Z. , Lu J. , Sun Z. , and Chen Z. , Redefining Bladder Cancer Treatment: Innovations in Overcoming Drug Resistance and Immune Evasion, Front Immunology. (2025) 16, no. 10364, 10.3389/fimmu.2025.1537808, 1537808. PMC11794230 39911393

[2] Bray F. , Laversanne M. , Sung H. , Ferlay J. , Siegel R. L. , Soerjomataram I. , and Jemal A. , Global Cancer Statistics 2022: GLOBOCAN Estimates of Incidence and Mortality Worldwide for 36 Cancers in 185 Countries, CA: A Cancer Journal for Clinicians. (2024) 74, no. 3, 229–263, 10.3322/caac.21834. 38572751

[3] Antoni S. , Ferlay J. , Soerjomataram I. , Znaor A. , Jemal A. , and Bray F. , Bladder Cancer Incidence and Mortality: A Global Overview and Recent Trends, European Urology. (2017) 71, no. 1, 96–108, 10.1016/j.eururo.2016.06.010, 2-s2.0-84979707958, 27370177. 27370177

[4] Compérat E. , Amin M. B. , Cathomas R. , Choudhury A. , De Santis M. , Kamat A. , Stenzl A. , Thoeny H. C. , and Witjes J. A. , Current Best Practice for Bladder Cancer: A Narrative Review of Diagnostics and Treatments, Lancet. (2022) 400, no. 10364, 1712–1721, 10.1016/S0140-6736(22)01188-6, 36174585. 36174585

[5] Lin L. , Hutzen B. , Zuo M. , Ball S. , Deangelis S. , Foust E. , Pandit B. , Ihnat M. A. , Shenoy S. S. , Kulp S. , Li P. K. , Li C. , Fuchs J. , and Lin J. , Novel STAT3 Phosphorylation Inhibitors Exhibit Potent Growth-Suppressive Activity in Pancreatic and Breast Cancer Cells, Cancer Research. (2010) 70, no. 6, 2445–2454, 10.1158/0008-5472.CAN-09-2468, 2-s2.0-77950190311, 20215512. 20215512 PMC2843552

[6] Zhang X. , Wang R. , Perez G. R. , Chen G. , Zhang Q. , Zheng S. , Wang G. , and Chen Q.-H. , Design, Synthesis, and Biological Evaluation of 1, 9-Diheteroarylnona-1, 3, 6, 8-Tetraen-5-Ones as a New Class of Anti-Prostate Cancer Agents, Bioorganic & Medicinal Chemistry. (2016) 24, no. 19, 4692–4700, 10.1016/j.bmc.2016.08.006, 2-s2.0-84985993364, 27543391. 27543391 PMC5014612

[7] Chiba T. , Mack L. , Delis N. , Brill B. , and Groner B. , Stat3 Inhibition in Neural Lineage Cells, Hormone Molecular Biology and Clinical Investigation. (2012) 10, no. 2, 255–263, 10.1515/hmbci-2012-0005, 2-s2.0-84979527473. 25436682

[8] Yuan S. , Cao S. , Jiang R. , Liu R. , Bai J. , and Hou Q. , FLLL31, a Derivative of Curcumin, Attenuates Airway Inflammation in a Multi-Allergen Challenged Mouse Model, International Immunopharmacology. (2014) 21, no. 1, 128–136, 10.1016/j.intimp.2014.04.020, 2-s2.0-84901060419, 24819716. 24819716

[9] Link W. , Introduction to FOXO biology, FOXO Transcription Factors: Methods and Protocols, 2019, Springer, 1–9, 10.1007/978-1-4939-8900-3_1, 2-s2.0-85056412496.

[10] Rodriguez-Colman M. J. , Dansen T. B. , and Burgering B. M. , FOXO Transcription Factors as Mediators of Stress Adaptation, Nature Reviews Molecular Cell Biology. (2024) 25, no. 1, 46–64, 10.1038/s41580-023-00649-0, 37710009. 37710009

[11] Wang Y. , Kang X.-L. , Zeng F.-C. , Xu C.-J. , Zhou J.-Q. , and Luo D.-N. , Correlations of Foxo3 and Foxo4 Expressions With Clinicopathological Features and Prognosis of Bladder Cancer, Pathology-Research and Practice. (2017) 213, no. 7, 766–772, 10.1016/j.prp.2017.04.004, 2-s2.0-85019838073, 28554751. 28554751

[12] Chen Y. , Zhu H. , Luo Y. , Xie T. , Hu Y. , Yan Z. , Ji W. , Wang Y. , Yin Q. , and Xian H. , ALDOC Promotes Neuroblastoma Progression and Modulates Sensitivity to Chemotherapy Drugs by Enhancing Aerobic Glycolysis, Frontiers in Immunology. (2025) 16, 1573815, 10.3389/fimmu.2025.1573815, 40313939. 40313939 PMC12043483

[13] Mi D. , Li C. , Li Y. , Yao M. , Li Y. , Hong K. , Xie C. , and Chen Y. , Discovery of Novel BCL6-Targeting PROTACs With Effective Antitumor Activities Against DLBCL *In Vitro* and *In Vivo* , European Journal of Medicinal Chemistry. (2024) 277, 116789, 10.1016/j.ejmech.2024.116789, 39208743. 39208743

[14] Wang Q. , Sztukowska M. , Ojo A. , Scott D. A. , Wang H. , and Lamont R. J. , FOXO Responses to *Porphyromonas gingivalis* in Epithelial Cells, Cellular Microbiology. (2015) 17, no. 11, 1605–1617, 10.1111/cmi.12459, 2-s2.0-84945306967, 25958948. 25958948 PMC4624012

[15] Nakae J. , Kitamura T. , Ogawa W. , Kasuga M. , and Accili D. , Insulin Regulation of Gene Expression Through the Forkhead Transcription Factor Foxo1 (Fkhr) Requires Kinases Distinct From Akt, Biochemistry. (2001) 40, no. 39, 11768–11776, 10.1021/bi015532m, 2-s2.0-0035797921, 11570877. 11570877

[16] Harrington J. S. , Ryter S. W. , Plataki M. , Price D. R. , and Choi A. M. K. , Mitochondria in Health, Disease, and Aging, Physiological Reviews. (2023) 103, no. 4, 2349–2422, 10.1152/physrev.00058.2021, 37021870. 37021870 PMC10393386

[17] Bock F. J. and Tait S. W. , Mitochondria as Multifaceted Regulators of Cell Death, Nature Reviews Molecular Cell Biology. (2020) 21, no. 2, 85–100, 10.1038/s41580-019-0173-8. 31636403

[18] Vringer E. and Tait S. W. , Mitochondria and Cell Death-Associated Inflammation, Cell Death & Differentiation. (2023) 30, no. 2, 304–312, 10.1038/s41418-022-01094-w, 36447047. 36447047 PMC9950460

[19] Renault T. T. , Dejean L. M. , and Manon S. , A Brewing Understanding of the Regulation of Bax Function by Bcl-xL and Bcl-2, Mechanisms of Ageing and Development. (2017) 161, pt. B, 201–210, 10.1016/j.mad.2016.04.007, 2-s2.0-84964652721, 27112371. 27112371

[20] Li M. , Wang D. , He J. , Chen L. , and Li H. , Bcl-X (L): A Multifunctional Anti-Apoptotic Protein, Pharmacological Research. (2020) 151, 104547, 10.1016/j.phrs.2019.104547, 31734345. 31734345

[21] Boise L. H. , González-García M. , Postema C. E. , Ding L. , Lindsten T. , Turka L. A. , Mao X. , Nuñez G. , and Thompson C. B. , Bcl-x, a Bcl-2-Related Gene That Functions as a Dominant Regulator of Apoptotic Cell Death, Cell. (1993) 74, no. 4, 597–608, 10.1016/0092-8674(93)90508-n, 2-s2.0-0027282044, 8358789. 8358789

[22] Huang K. , O′Neill K. L. , Li J. , Zhou W. , Han N. , Pang X. , Wu W. , Struble L. , Borgstahl G. , Liu Z. , Zhang L. , and Luo X. , BH3-Only Proteins Target BCL-xL/MCL-1, Not BAX/BAK, to Initiate Apoptosis, Cell Research. (2019) 29, no. 11, 942–952, 10.1038/s41422-019-0231-y, 2-s2.0-85073945686, 31551537. 31551537 PMC6888900

[23] Czabotar P. E. , Westphal D. , Dewson G. , Ma S. , Hockings C. , Fairlie W. D. , Lee E. F. , Yao S. , Robin A. Y. , Smith B. J. , Huang D. C. , Kluck R. M. , Adams J. M. , and Colman P. M. , Bax Crystal Structures Reveal How BH3 Domains Activate Bax and Nucleate Its Oligomerization to Induce Apoptosis, Cell. (2013) 152, no. 3, 519–531, 10.1016/j.cell.2012.12.031, 2-s2.0-84873307384, 23374347. 23374347

[24] Burke P. J. , Mitochondria, Bioenergetics and Apoptosis in Cancer, Trends in Cancer. (2017) 3, no. 12, 857–870, 10.1016/j.trecan.2017.10.006, 2-s2.0-85034758163. 29198441 PMC5957506

[25] Soler-Agesta R. , Anel A. , and Galluzzi L. , Mitochondrial Control of Antigen Presentation in Cancer Cells, Cancer Cell. (2023) 41, no. 11, 1849–1851, 10.1016/j.ccell.2023.10.001, 37890494. 37890494

[26] Wise A. R. , Maloney S. , Hering A. , Zabala S. , Richmond G. E. , VanKlompenberg M. K. , Nair M. T. , and Prosperi J. R. , Bcl-2 Up-Regulation Mediates Taxane Resistance Downstream of APC Loss, International Journal of Molecular Sciences. (2024) 25, no. 12, 10.3390/ijms25126745, 38928449. PMC11203545 38928449

[27] Mo L.-H. , Han H.-Y. , Jin Q.-R. , Song Y.-N. , Wu G.-H. , Zhang Y. , Yang L.-T. , Liu T. , Liu Z.-G. , Feng Y. , and Yang P. C. , T Cell Activator-Carrying Extracellular Vesicles Induce Antigen-Specific Regulatory T Cells, Clinical & Experimental Immunology. (2021) 206, no. 2, 129–140, 10.1111/cei.13655, 34418066. 34418066 PMC8506129

[28] Kuusanmäki H. , Dufva O. , Vähä-Koskela M. , Leppä A.-M. , Huuhtanen J. , Vänttinen I. , Nygren P. , Klievink J. , Bouhlal J. , Pölönen P. , Zhang Q. , Adnan-Awad S. , Mancebo-Pérez C. , Saad J. , Miettinen J. , Javarappa K. K. , Aakko S. , Ruokoranta T. , Eldfors S. , Heinäniemi M. , Theilgaard-Mönch K. , Wartiovaara-Kautto U. , Keränen M. , Porkka K. , Konopleva M. , Wennerberg K. , Kontro M. , Heckman C. A. , and Mustjoki S. , Erythroid/Megakaryocytic Differentiation Confers BCL-XL Dependency and Venetoclax Resistance in Acute Myeloid Leukemia, Blood. (2023) 141, no. 13, 1610–1625, 10.1182/blood.2021011094, 36508699. 36508699 PMC10651789

[29] Yamochi T. , Kaneita Y. , Akiyama T. , Mori S. , and Moriyama M. , Adenovirus-Mediated High Expression of BCL-6 in CV-1 Cells Induces Apoptotic Cell Death Accompanied by Down-Regulation of BCL-2 and BCL-X_L_ , Oncogene. (1999) 18, no. 2, 487–494, 10.1038/sj.onc.1202334, 2-s2.0-0033552949, 9927205. 9927205

[30] Vogler M. , Braun Y. , Smith V. M. , Westhoff M. A. , Pereira R. S. , Pieper N. M. , Anders M. , Callens M. , Vervliet T. , Abbas M. , Macip S. , Schmid R. , Bultynck G. , and Dyer M. J. , The BCL2 Family: From Apoptosis Mechanisms to New Advances in Targeted Therapy, Signal Transduction and Targeted Therapy. (2025) 10, no. 1, 10.1038/s41392-025-02176-0, 40113751. PMC11926181 40113751

[31] Lin L. , Hutzen B. , Ball S. , Foust E. , Sobo M. , Deangelis S. , Pandit B. , Friedman L. , Li C. , Li P. K. , Fuchs J. , and Lin J. , New Curcumin Analogues Exhibit Enhanced Growth-Suppressive Activity and Inhibit AKT and Signal Transducer and Activator of Transcription 3 Phosphorylation in Breast and Prostate Cancer Cells, Cancer Science. (2009) 100, no. 9, 1719–1727, 10.1111/j.1349-7006.2009.01220.x, 2-s2.0-70149085608, 19558577. 19558577 PMC11158315

[32] Kohoutova K. , Srb P. , Obsilova V. , Veverka V. , and Obsil T. , Structural Plasticity of the FOXO-DBD: p53-TAD Interaction, Nature Communications. (2025) 16, no. 1, 10.1038/s41467-025-59106-5, 40425537. PMC12117093 40425537

[33] Su B. , Gao L. , Baranowski C. , Gillard B. , Wang J. , Ransom R. , Ko H. K. , and Gelman I. H. , A Genome-Wide RNAi Screen Identifies FOXO4 as a Metastasis-Suppressor Through Counteracting PI3K/AKT Signal Pathway in Prostate Cancer, PLoS One. (2014) 9, no. 7, e101411, 10.1371/journal.pone.0101411, 2-s2.0-84903626213, 24983969. 24983969 PMC4077825

[34] Zhong T. , Li Y. , Jin M. , Liu J. , Wu Z. , Zhu F. , Zhao L. , Fan Y. , Xu L. , and Ji J. , Downregulation of 4-HNE and FOXO4 Collaboratively Promotes NSCLC Cell Migration and Tumor Growth, Cell Death & Disease. (2024) 15, no. 7, 10.1038/s41419-024-06948-4, 39085238. PMC11291900 39085238

[35] Tang T. T. L. , Dowbenko D. , Jackson A. , Toney L. , Lewin D. A. , Dent A. L. , and Lasky L. A. , The Forkhead Transcription Factor AFX Activates Apoptosis by Induction of the BCL-6 Transcriptional Repressor, Journal of Biological Chemistry. (2002) 277, no. 16, 14255–14265, 10.1074/jbc.M110901200, 2-s2.0-0037134491, 11777915. 11777915

[36] Bertheloot D. , Latz E. , and Franklin B. S. , Necroptosis, Pyroptosis and Apoptosis: An Intricate Game of Cell Death, Cellular & Molecular Immunology. (2021) 18, no. 5, 1106–1121, 10.1038/s41423-020-00630-3, 33785842. 33785842 PMC8008022

[37] Yuan J. and Ofengeim D. , A Guide to Cell Death Pathways, Nature Reviews Molecular Cell Biology. (2024) 25, no. 5, 379–395, 10.1038/s41580-023-00689-6. 38110635

[38] Fleisher T. A. , Apoptosis, Annals of Allergy, Asthma & Immunology. (1997) 78, no. 3, 245–250, 10.1016/S1081-1206(10)63176-6, 2-s2.0-0030956688. 9087147

[39] Moyer A. , Tanaka K. , and Cheng E. H. , Apoptosis in Cancer Biology and Therapy, Annual Review of Pathology. (2025) 20, no. 1, 303–328, 10.1146/annurev-pathmechdis-051222-115023. 39854189

[40] Wang X. H. , Jiang Z. H. , Yang H. M. , Zhang Y. , and Xu L. H. , Hypoxia-Induced FOXO4/LDHA Axis Modulates Gastric Cancer Cell Glycolysis and Progression, Clinical and Translational Medicine. (2021) 11, no. 1, e279, 10.1002/ctm2.279, 33463054. 33463054 PMC7809603

[41] Sun Y. , Wang L. , Xu X. , Han P. , Wu J. , Tian X. , and Li M. , FOXO4 Inhibits the Migration and Metastasis of Colorectal Cancer by Regulating the APC2/*β*-Catenin Axis, Frontiers in Cell and Developmental Biology. (2021) 9, 659731, 10.3389/fcell.2021.659731, 34631691. 34631691 PMC8495124

[42] Cardenas M. G. , Oswald E. , Yu W. , Xue F. , MacKerell A. D.Jr., and Melnick A. M. , The Expanding Role of the BCL6 Oncoprotein as a Cancer Therapeutic Target, Clinical Cancer Research. (2017) 23, no. 4, 885–893, 10.1158/1078-0432.CCR-16-2071, 2-s2.0-85012921549, 27881582. 27881582 PMC5315622

[43] Li K. , Liu Y. , Ding Y. , Zhang Z. , Feng J. , Hu J. , Chen J. , Lian Z. , Chen Y. , Hu K. , Chen Z. , Cai Z. , Liu M. , and Pang X. , BCL6 is Regulated by the MAPK/ELK1 Axis and Promotes KRAS-Driven Lung Cancer, Journal of Clinical Investigation. (2022) 132, no. 22, 10.1172/JCI161308, 36377663. PMC9663163 36377663

[44] Sarott R. C. , Gourisankar S. , Karim B. , Nettles S. , Yang H. , Dwyer B. G. , Simanauskaite J. M. , Tse J. , Abuzaid H. , Krokhotin A. , Zhang T. , Hinshaw S. M. , Green M. R. , Crabtree G. R. , and Gray N. S. , Relocalizing Transcriptional Kinases to Activate Apoptosis, Science. (2024) 386, no. 6717, eadl5361, 10.1126/science.adl5361, 39361741. 39361741 PMC11629774

[45] Phan R. T. and Dalla-Favera R. , The *BCL6* Proto-Oncogene Suppresses p53 Expression in Germinal-Centre B Cells, Nature. (2004) 432, no. 7017, 635–639, 10.1038/nature03147, 2-s2.0-10344247666. 15577913

[46] Wang Y. , Zhu H. , Ren J. , and Ren M. , Integrative Machine Learning Models Predict Prostate Cancer Diagnosis and Biochemical Recurrence Risk: Advancing Precision Oncology, NPJ Digital Medicine. (2025) 8, no. 1, 10.1038/s41746-025-01930-6, 40819002. PMC12357910 40819002

